# Microbial Transcriptional and Metabolic Shifts Mediate Rhizosphere Organic Carbon Accumulation in Alpine Grasslands Following Six Years of Continuous Nitrogen Addition

**DOI:** 10.3390/microorganisms14081808

**Published:** 2026-08-17

**Authors:** Zheng Wu, Lingchen Tong, Wenqiang Huang, Minghang Hu, Shuang Liu, Yanying Han, Guangyu Zhang, Yanhui Ye

**Affiliations:** 1 College of Forestry and Grassland, Xizang Agricultural and Animal Husbandry University, Nyingchi 860000, China; humuou@163.com (Z.W.); tlc2317516@163.com (L.T.); wenqianghuang66@gmail.com (W.H.);; 2 Key Laboratory of Forest Ecology in Xizang Plateau, Ministry of Education, Xizang Agricultural and Animal Husbandry University, Nyingchi 860000, China; 3 Institute of Geographic Sciences and Natural Resources Research, Chinese Academy of Sciences, Beijing 100101, China; 4 Lhasa Plateau Ecosystem Research Station, Key Laboratory of Ecosystem Network Observation and Modeling, Institute of Geographic Sciences and Natural Resources Research, Lhasa 850000, China; 5 Beijing Key Laboratory of Biodiversity and Organic Agriculture, China Agricultural University, Beijing 100193, China

**Keywords:** nitrogen addition, alpine meadows, metatranscriptomics, rhizosphere metabolites, soil organic carbon, microbial community, threshold effect

## Abstract

Global nitrogen (N) deposition alters soil organic carbon (SOC) dynamics in alpine grasslands, yet rhizosphere carbon turnover mechanisms remain debated due to a lack of integrated multi-omics insights. This study aimed to systematically investigate the rhizosphere carbon turnover mechanisms and explore the 6-year N addition thresholds in alpine grasslands. Leveraging a six-year in situ N addition experiment, we combined metatranscriptomics and untargeted metabolomics to systematically investigate rhizosphere carbon pathways. The 10 kg N ha^−1^ yr^−1^ treatment (N10) alleviated nutrient limitation by increasing microbial biomass nitrogen and dissolved organic nitrogen. This significantly upregulated carbon fixation genes, particularly the reductive tricarboxylic acid (rTCA) cycle, promoting the accumulation of lipids and organic acids, which subsequently increased total SOC, particulate organic carbon (POC), and mineral-associated organic carbon (MAOC). Conversely, when N inputs exceeded this threshold (e.g., the 20 kg N ha^−1^ yr^−1^ treatment), the additions induced severe soil acidification, suppressed carbon fixation transcription, and shifted the rhizosphere toward a degradation-dominated state, decreasing key carbonaceous metabolites and reducing all organic carbon fractions. Overall, continuous N input exerts a significant nonlinear threshold effect on rhizosphere carbon sequestration. Sustained N loading beyond the ecological threshold undermines soil carbon pool stability through coupled physicochemical, transcriptional, and metabolic alterations, highlighting the severe ecological risks of continuous N deposition in alpine ecosystems.

## 1. Introduction

Globally, increased anthropogenic inputs of reactive nitrogen (N) have fundamentally altered the biogeochemical cycles of terrestrial ecosystems [1,2,3]. Recent estimates indicate that global anthropogenic N deposition has approximately tripled compared to historical baselines; by 2020, accumulated anthropogenic reactive N had reached 226 million tons, vastly exceeding natural N fixation rates [1,2,3]. Serving as a sensitive indicator region for global climate change, alpine grasslands on the Qinghai–Tibet Plateau have long been constrained by low temperatures and severe N limitation [4,5]. Under these environmental conditions, soil microbial metabolic activity is restricted, and organic matter decomposition proceeds slowly. This dynamic facilitates the belowground accumulation and sequestration of a massive soil organic carbon (SOC) pool, which accounts for approximately 2.5% of the global terrestrial carbon reservoir [6,7,8]. Given their limited systemic buffering capacity, alpine meadows are highly sensitive to exogenous nutrient inputs. The introduction of exogenous N disrupts the inherent stoichiometric balance of the ecosystem, thereby triggering cascading effects across belowground ecological processes [9].

Conclusions regarding how N addition reshapes rhizosphere carbon turnover in alpine meadows remain debated. Some studies indicate that N inputs alleviate N limitation in alpine ecosystems and stimulate rhizosphere microbiomes by enhancing the input of root exudates, such as organic acids [10]. This process can increase microbial carbon use efficiency (CUE) by 11.5–25.5%, ultimately promoting rhizosphere carbon sequestration [11,12]. Conversely, alternative research highlights that sustained N enrichment drastically lowers soil pH, driving the deterioration of rhizosphere microenvironments [13]. Such severe physicochemical shifts not only diminish microbial biomass carbon (MBC) [14], but also alter the decomposition dynamics of recalcitrant organic matter. Specifically, these shifts upregulate carbon-degrading enzyme pathways, including Auxiliary Activities (AAs) for lignin oxidation and Glycoside Hydrolases (GHs) for polysaccharide degradation [15]. This upregulation ultimately accelerates SOC mineralization and release [16,17]. Consequently, we still lack a systematic consensus on how continuous N inputs modulate these key carbon turnover pathways, particularly regarding the dynamic balance between carbon fixation and degradation within the rhizosphere.

Traditional studies have predominantly focused on bulk soil and bulk organic carbon analyses, frequently overlooking the central role of the rhizosphere microenvironment in biogeochemical cycling [18,19,20]. Consequently, mechanistic insights into physical carbon fractions within the rhizosphere remain limited [9,21,22]. Furthermore, mechanistic investigations of grassland carbon turnover under environmental disturbances have heavily relied on microbial community profiling or DNA-based assessments of functional potential [23,24]. However, the transcription of soil carbon-cycling genes (RNA) can exhibit dramatic short-term fluctuations following nutrient addition [25,26]. This transcriptional sensitivity more accurately reflects the real-time functional activity of the microbiome [27]. More importantly, the transcriptional expression of rhizosphere microorganisms ultimately manifests in the metabolite profile [28]. These metabolites serve as the fundamental precursors for the formation and maintenance of stable carbon pools via mechanisms such as the microbial carbon pump (MCP) [29,30]. Consequently, current research on alpine grassland carbon turnover is largely confined to single-omics approaches. A critical gap remains for integrated multi-omics analyses that explicitly link active transcription, active metabolites, and physical SOC fractions [31,32].

To elucidate the transcriptional and metabolic shifts underlying rhizosphere carbon turnover in alpine meadows following six consecutive years of N input, this study leveraged an in situ N addition gradient experiment on the western slope of Sejila Mountain in Nyingchi, Tibet. Given the local background N deposition (approximately 5.17 kg N ha^−1^ yr^−1^) and projected future scenarios for the Qinghai–Tibet Plateau, this explicit 6-year continuous N addition gradient (0, 10, 15, and 20 kg N ha^−1^ yr^−1^) was designed to precisely identify the critical ecological threshold at which positive stimulation of rhizosphere functions transitions into ecosystem degradation. By integrating metatranscriptomics and untargeted metabolomics, we systematically investigated a cascading response framework encompassing N addition gradients, environmental shifts, microbial transcriptional reprogramming, metabolic alterations, and subsequent dynamics of physical SOC fractions. Based on this framework, we tested the following three hypotheses:

**H1.**  *The N10 treatment alleviates nutrient limitation, promoting the accumulation of total SOC and its physical fractions—namely, particulate and mineral-associated organic carbon (POC and MAOC). Conversely, severe soil physicochemical shifts induced by N inputs exceeding 10 kg N ha^−1^ yr^−1^ drive rhizosphere carbon loss.*

**H2.**  *The N10 treatment upregulates the transcription of specific carbon fixation genes, whereas soil acidification under elevated N inputs (>10 kg N ha^−1^ yr^−1^) suppresses carbon fixation transcription, thereby shifting rhizosphere carbon turnover toward a degradation-dominated regime.*

**H3.**  *By reshaping the transcriptional balance between carbon fixation and degradation, N addition alters key rhizosphere metabolite profiles, which ultimately dictates the mechanistic trajectory of POC and MAOC—determining whether the system shifts toward carbon accumulation or loss.*

## 2. Materials and Methods

### 2.1. Study Site and Experimental Design

The in situ-controlled field experiment was established on the western slope of Sejila Mountain in Nyingchi, Tibet Autonomous Region, China (29°37′ N, 94°37′ E; elevation ~4400 m a.s.l.). This region features a montane temperate humid and sub-humid climate characterized by severe environmental conditions. The mean annual temperature (MAT) is approximately −0.73 °C, and the mean annual precipitation (MAP) is 1267.2 mm [33,34], which predominantly occurs during the plant-growing season (April to October). The frost-free period spans approximately 180 days, with relative humidity ranging between 60% and 80%. The soil is classified as a typical alpine meadow soil. The aboveground plant community is dominated by perennial herbaceous species, primarily *Elymus dahuricus*, *Kobresia royleana*, and *Bistorta vivipara*.

The continuous N addition experiment was initiated in May 2019 using a randomized complete block design. Accounting for the local background N deposition flux (5.17 kg N ha^−1^ yr^−1^) [33] and simulating projected increases in future atmospheric N deposition, we established four N input gradient levels: an unamended control (CK, 0 kg N ha^−1^ yr^−1^) and three N addition treatments (10, 15, and 20 kg N ha^−1^ yr^−1^, designated as N10, N15, and N20, respectively). Twelve independent 9-m^2^ (3 m × 3 m) plots were randomly assigned across three blocks. To prevent cross-contamination, 1-m and 5-m buffer zones separated adjacent plots and blocks, respectively. Additionally, the entire experimental perimeter was fenced to exclude large grazing herbivores. N addition was conducted annually in mid-August, immediately following the annual soil sample collection. To control for the confounding effects of water addition, CK plots concurrently received an equal volume of purified water. For the current study, the precise soil sampling campaign was executed in mid-August 2025, a period coinciding with the peak growing season of the alpine meadow when root exudation and microbial metabolic activities are highly robust. At the time of sampling for the current study, these gradient N addition treatments had been continuously maintained for six years.

### 2.2. Soil Sampling and Processing

Soil sampling specifically targeted the rhizosphere. Within each N addition plot, five sampling points were selected using a standard quincunx spatial pattern. A soil auger (5-cm inner diameter) was used to excavate intact, mixed-community plant root systems from the 0–20 cm soil profile. Following excavation, the loose, unattached bulk soil was gently shaken off the intact root systems. Subsequently, under sterile conditions, the soil adhering strictly within 1–2 mm of the root surface was carefully collected using a nylon brush; this specific fraction was defined as the rhizosphere soil [35].

Rhizosphere soil from the five sampling points within a single plot was rapidly pooled and homogenized in the field, then immediately passed through a sterile 2-mm mesh sieve to remove gravel, visible plant roots, and animal debris. This homogenized composite sample constituted a single biological replicate for that plot. The entire experiment comprised 12 independent plots in total, yielding 3 biological replicates for each N treatment. Each processed rhizosphere composite sample was promptly divided into two aliquots. The first aliquot was flash-frozen in liquid N, transported to the laboratory, and stored at −80 °C for metatranscriptomic and untargeted metabolomic sequencing. The second aliquot was temporarily stored at 4 °C in a portable cooler to ensure that basic soil physicochemical parameters were determined within one week. Densely interwoven fine roots and overlapping rhizosphere zones collectively constitute a complex intertwined rhizosphere network [35,36]. Therefore, we collected mixed-community rhizosphere soil samples to evaluate the integrated ecosystem-level responses to the 6-year N addition.

### 2.3. Determination of Soil Physicochemical Properties

Soil pH was measured in a 1:2.5 (*w*/*v*) soil-to-water suspension. The SOC and total nitrogen (TN) contents were quantified via high-temperature dry combustion using an elemental analyzer (EA 3000; Elementar, Langenselbold, Germany). The physical fractions of SOC, specifically POC and MAOC, were separated via a wet sieving method. Briefly, 10 g of air-dried soil was dispersed in a 5 g L^−1^ sodium hexametaphosphate solution and shaken to thoroughly disrupt soil aggregates. The suspension was then wet-sieved through a 53-μm mesh. The coarse fraction retained on the sieve (>53 μm) and the fine fraction passing through (<53 μm) were separately collected, dried, and analyzed for C content using the aforementioned elemental analyzer to determine POC and MAOC, respectively.

Inorganic N fractions (ammonium, NH_4_^+^-N; and nitrate, NO_3_^−^-N) were extracted from fresh soil samples using a 2 mol L^−1^ KCl solution and quantified colorimetrically on an automated flow injection analyzer (AACE; SEAL Analytical, Norderstedt, Germany). Dissolved organic carbon (DOC) and total dissolved nitrogen (TDN) were extracted using a 0.5 mol L^−1^ K_2_SO_4_ solution, filtered, and analyzed on a TOC/TN analyzer (multi N/C 3100; Analytik Jena, Jena, Germany). Dissolved organic nitrogen (DON) was subsequently calculated as the difference between TDN and total inorganic N [37,38]. MBC and microbial biomass nitrogen (MBN) were determined using the chloroform fumigation-extraction method, utilizing 0.5 mol L^−1^ K_2_SO_4_ as the extractant. Biomass values were calculated based on the differences in extractable C and N between fumigated and unfumigated paired samples, employing extraction efficiency conversion factors of *k*_EC_ = 0.45 and *k*_EN_ = 0.54, respectively [39].

### 2.4. Metatranscriptomic Sequencing and Bioinformatic Processing

Total RNA was extracted from the rhizosphere soil using the Soil RNA Extraction Kit (Majorbio, Shanghai, China). Crucially, an on-column DNase I digestion step was incorporated to strictly eliminate any residual genomic DNA contamination. Following RNA integrity evaluation (RQN > 7.0), ribosomal RNA (rRNA) was depleted using the RiboCop rRNA Depletion Kit (Lexogen, Greenland, NH, USA). Subsequently, strand-specific cDNA libraries were constructed. Following library quality control, DNA nanoballs (DNBs) were generated via rolling circle amplification (RCA). Deep sequencing was then performed on the DNBSEQ-T7 platform (BGI, Shenzhen, China) utilizing a 150-bp paired-end (PE150) sequencing strategy [40]. To ensure comprehensive capture of the active rhizosphere microbiome, each sample was sequenced to generate approximately 6 Gb of raw data.

Raw sequence data were quality-filtered using fastp (v0.23.1, parameter: --cut_mean_quality 30) [41], and SortMeRNA was applied to rigorously eliminate residual rRNA fragments. The resulting high-quality clean reads were subjected to de novo assembly using Trinity (v2.15.1, --min_contig_length 200). Trinity was specifically selected for its robustness in handling the highly uneven coverage of environmental microbiomes and its ability to co-assemble mixed communities containing both prokaryotic and eukaryotic (e.g., fungal) transcripts. To mitigate potential assembly-induced redundancy (such as eukaryotic isoforms or prokaryotic strain variations) and construct a high-fidelity non-redundant gene catalog, the assembled transcripts were strictly collapsed utilizing CD-HIT-EST with a sequence identity threshold of 95% (-c 0.95). Finally, the clean reads were mapped back against this non-redundant gene catalog, and transcript abundances were quantified using RSEM (v1.3.1, --paired-end --bowtie2) [42]. Transcript expression levels were normalized to Transcripts Per Kilobase Million (TPM) to calculate the relative abundance of core metabolic pathways for subsequent ecological and statistical comparisons [43].

### 2.5. Functional Annotation of Key Carbon Turnover Pathways

To investigate N-driven carbon sequestration and degradation pathways in the alpine grassland ecosystem, the non-redundant gene catalog was annotated against public databases utilizing eggNOG-mapper (core parameters: -m diamond --translate --seed_ortholog_evalue 1 × 10^−5^) [44]. To explicitly address our core research objectives, we primarily utilized the KEGG database (https://www.genome.jp/kegg/, accessed on 25 February 2026) for carbon fixation pathways and the CAZy database (http://www.cazy.org/, accessed on 25 February 2026) for carbon degradation networks [45,46]. Specifically, by aligning sequences against the KEGG database [45], core orthologous genes mediating microbial dark carbon fixation were extracted and quantified. These transcript abundances were precisely mapped to five fundamental carbon fixation pathways: the Calvin–Benson–Bassham (CBB) cycle, the reductive tricarboxylic acid (*rTCA*) cycle, the Wood–Ljungdahl (WL) pathway, the 3-hydroxypropionate (3-HP) bicycle, and the 4-hydroxybutyrate (4-HB) cycle [47].

Simultaneously, to comprehensively profile the organic carbon-degrading enzyme systems, sequences were queried against the CAZy database [46]. This targeted alignment systematically quantified the transcriptional abundance shifts in core enzyme families, specifically encompassing Glycoside Hydrolases (GHs) responsible for cellulose and hemicellulose degradation, Auxiliary Activities (AAs) involved in lignin oxidation, Carbohydrate Esterases (CEs) involved in polysaccharide modification, and Polysaccharide Lyases (PLs) responsible for pectin cleavage.

### 2.6. Untargeted Metabolomic Analysis

Briefly, 100 mg of freeze-dried rhizosphere soil was precisely weighed and subjected to extraction utilizing a ternary solvent mixture comprising methanol, acetonitrile, and ultrapure water (2:2:1, *v*/*v*/*v*). The mixture underwent three consecutive extraction cycles, with each cycle consisting of vortexing (30 s), mechanical tissue grinding (35 Hz, 4 min), and ultrasonication in an ice bath (5 min). Extracts were subsequently incubated at −40 °C for 1 h and centrifuged at a low temperature (12,000 rpm, 15 min). The resulting supernatant was collected and analyzed via ultra-high-performance liquid chromatography coupled to high-resolution mass spectrometry (UHPLC-HRMS; Orbitrap Exploris™ 480, Thermo Fisher Scientific, Waltham, MA, USA) operating in both positive and negative ion modes. Quality control (QC) samples were systematically interspersed throughout the analytical sequence to continuously monitor instrument stability. Raw MS spectra were converted to mzXML format utilizing ProteoWizard. Peak detection, alignment, and retention time correction were executed using the xcms package in R [48]. Metabolic features exhibiting a missing value rate > 50% across biological samples or a coefficient of variation (CV) > 30% in QC samples were rigidly excluded [49]. To mitigate systematic bias and fulfill the strict prerequisite assumptions for multivariate statistical modeling, the filtered dataset was sequentially normalized to the median sample abundance, log-transformed to approximate a normal distribution, and subjected to auto-scaling [50].

Successfully annotated metabolites were cross-referenced against the Human Metabolome Database (HMDB) and ClassyFire databases. These were strictly categorized into 10 fundamental ecochemical superclasses [51]: lipids and lipid-like molecules; organic acids and derivatives; organoheterocyclic compounds; organic oxygen compounds; organic nitrogen compounds; phenylpropanoids and polyketides; benzenoids; nucleosides, nucleotides, and analogues; alkaloids and derivatives; and lignans, neolignans, and related compounds.

### 2.7. Statistical and Network Analyses

All statistical analyses and data visualizations were executed within the R statistical environment (v4.5.3) [52]. One-way analysis of variance (ANOVA) followed by Tukey’s honestly significant difference (HSD) post hoc test was utilized to evaluate significant differences among the N addition treatments (*p* < 0.05). Prior to multivariate modeling, all continuous variables were standardized via Z-score transformations.

A Mantel test, based on Spearman’s rank correlation coefficients, was conducted to assess the specific associations between soil physicochemical factors and core carbon-cycling transcriptional pathways, with the statistical linkages visualized as correlation networks [53]. For the untargeted metabolomic data, principal coordinate analysis (PCoA) coupled with permutational multivariate analysis of variance (PERMANOVA) was employed to evaluate compositional shifts in the overall metabolome structure. Partial least squares-discriminant analysis (PLS-DA) was applied to robustly screen for significantly altered metabolites, while principal component analysis (PCA) was integrated to identify the core driver metabolic superclasses across the N input gradient. Statistical associations between these differential metabolites and transcriptional activities were established via Spearman’s correlation analysis.

Finally, piecewise structural equation modeling (pSEM) was constructed utilizing the piecewiseSEM package (v2.3.1) [54]. This aimed to mechanistically elucidate the cascading pathways through which continuous N addition alters soil pH, subsequently shifting gene transcription and metabolic profiles, and ultimately driving the dynamics of physical SOC fractions. The overall goodness-of-fit for the model was rigorously evaluated using the directed separation (*d*-separation) criterion and Fisher’s *C* statistic (*p* > 0.05), with the final topology optimized via the Akaike Information Criterion (AIC) [55].

## 3. Results

### 3.1. Effects of Continuous N Addition on Rhizosphere Physicochemical Properties and C/N Fractions

**Figure 1:**
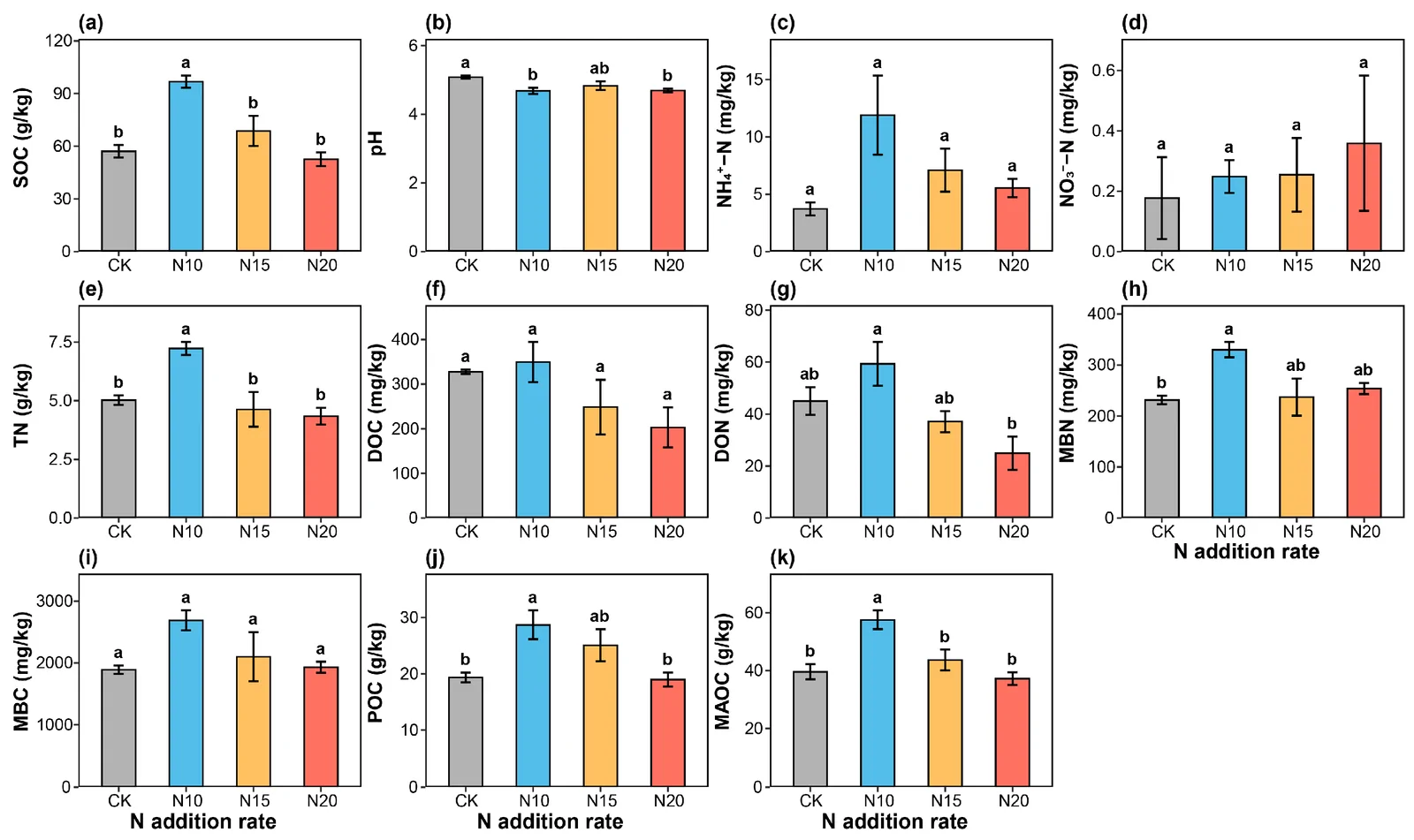
Effects of continuous six-year N addition on rhizosphere physicochemical properties and C/N fractions. Bar charts show responses across N input gradients (CK: 0, N10: 10, N15: 15, and N20: 20 kg N ha^−1^ yr^−1^) for: (**a**) soil organic carbon (SOC); (**b**) pH; (**c**) ammonium (NH_4_^+^-N); (**d**) nitrate (NO_3_^−^-N); (**e**) total nitrogen (TN); (**f**) dissolved organic carbon (DOC); (**g**) dissolved organic nitrogen (DON); (**h**) microbial biomass nitrogen (MBN); (**i**) microbial biomass carbon (MBC); (**j**) particulate organic carbon (POC); and (**k**) mineral-associated organic carbon (MAOC). Error bars indicate standard errors (SE, *n* = 3). Different lowercase letters denote significant differences among treatments (Tukey’s HSD test, *p* < 0.05).

Continuous six-year exogenous N addition significantly altered the physicochemical microenvironment of the rhizosphere (Figure 1). Compared to the unamended control (CK), soil pH decreased significantly under both the N10 and N20 treatments (*p* < 0.05). TN exhibited a pronounced nonlinear response across the N input gradient; it peaked in the N10 treatment, increasing significantly by 43.8% relative to CK, before sharply declining under the N15 and N20 treatments (*p* < 0.05). Within the inorganic N pool, NH_4_^+^-N displayed a non-significant unimodal trend (an initial increase followed by a decrease), whereas NO_3_^−^-N concentrations showed a marginal but statistically non-significant upward trajectory (*p* > 0.05).

Regarding the labile biological pools, the N10 treatment significantly enhanced MBN, yielding a 42.6% increase compared to CK (*p* < 0.05, Figure 1). DON also reached its maximum concentration under the N10 treatment, which was significantly higher than that of the N20 treatment (*p* < 0.05), although not significantly different from CK. In contrast, DOC and MBC contents remained relatively stable, with no statistically significant variations observed across the N addition gradient (*p* > 0.05).

Total SOC and its physical fractions exhibited consistent nonlinear accumulation dynamics in response to varying N inputs. The N10 treatment triggered a substantial carbon sequestration response, elevating the total SOC content by 69.5% relative to the control (*p* < 0.05, Figure 1). At the N10 threshold, both POC and MAOC increased synchronously to reach their respective maxima, significantly surpassing CK levels by 48.3% and 45.3%, respectively (*p* < 0.05). However, under the N15 and N20 input, the contents of SOC, POC, and MAOC rapidly collapsed to baseline levels, exhibiting no significant differences from the unamended control (*p* < 0.05).

### 3.2. Transcriptional Responses of Carbon Fixation and Degradation Genes and Contributions of Dominant Microbial Taxa

**Figure 2:**
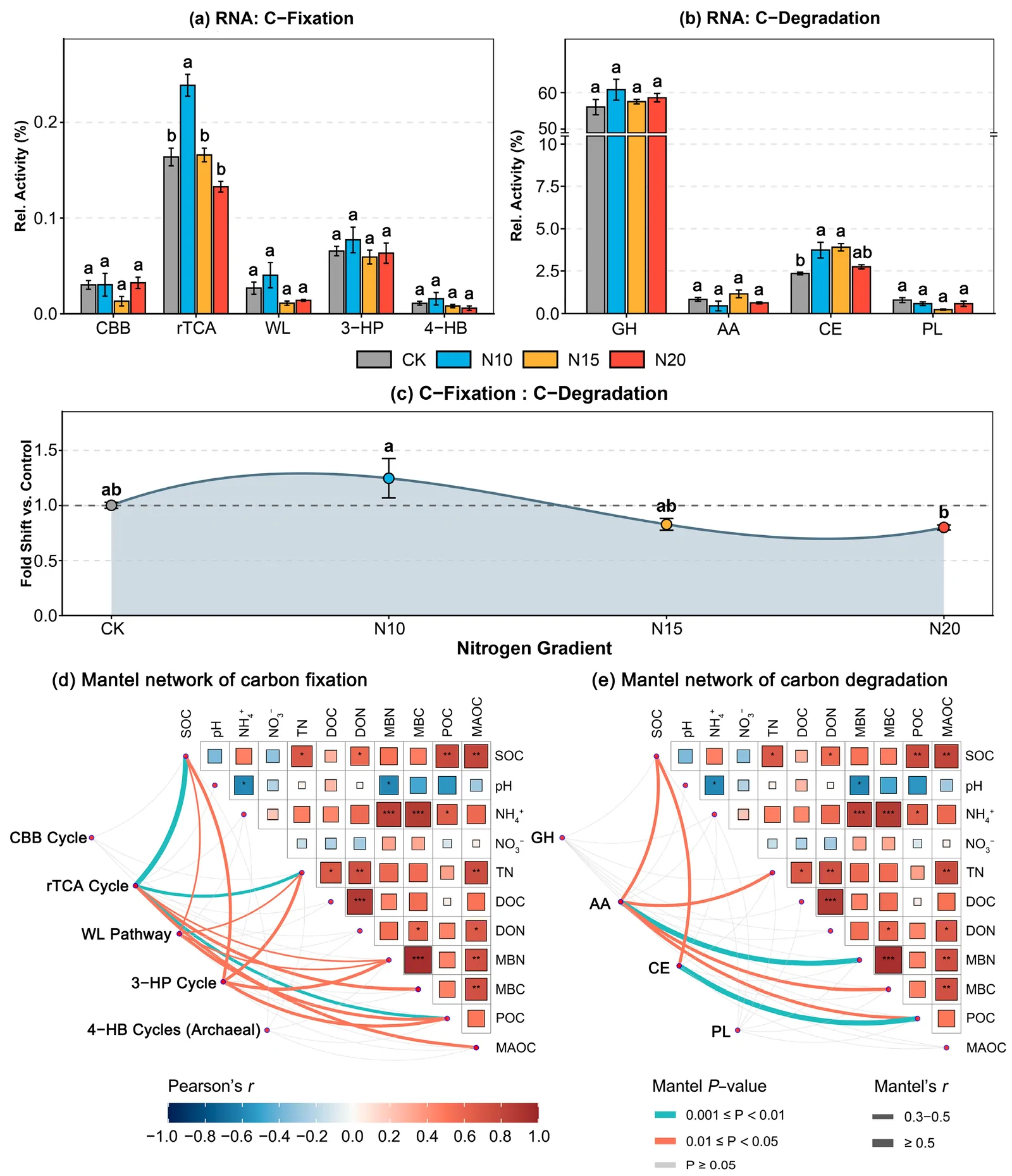
Transcriptional dynamics and environmental drivers of rhizosphere carbon cycling pathways under continuous six-year nitrogen (N) addition. (**a**,**b**) Relative transcriptional activities of core carbon fixation (**a**) and degradation (**b**) pathways. (**c**) Ratio of total fixation to degradation transcription. Lowercase letters denote significant differences among treatments within each pathway (one-way ANOVA with Tukey’s HSD, *p* < 0.05). (**d**,**e**) Mantel networks linking soil physicochemical properties with fixation (**d**) and degradation (**e**) pathways. Edge width and color indicate Mantel’s *r* and significance; heatmap colors reflect Pearson correlations (* *p* < 0.05, ** *p* < 0.01, and *** *p* < 0.001). N input rates: CK (0), N10 (10), N15 (15), and N20 (20) kg N ha^−1^ yr^−1^. Abbreviations: CBB, Calvin–Benson–Bassham; *rTCA*, reductive tricarboxylic acid; WL, Wood–Ljungdahl; 3-HP/4-HB, 3-hydroxypropionate/4-hydroxybutyrate; GH/CE/AA/PL, glycoside hydrolase/carbohydrate esterase/auxiliary activities/polysaccharide lyase; SOC/POC/MAOC, total/particulate/mineral-associated organic carbon; TN, total nitrogen; DOC/DON, dissolved organic carbon/nitrogen; MBC/MBN, microbial biomass carbon/nitrogen.

The transcriptional activity of rhizosphere carbon turnover pathways exhibited pronounced nonlinear dynamics across the N input gradient. Regarding carbon fixation, the transcriptional abundance of the *rTCA* cycle peaked under the N10 treatment, significantly exceeding that of the CK, N15, and N20 treatments (*p* < 0.05, Figure 2a). Other carbon fixation pathways remained statistically unaffected (*p* > 0.05). Concerning carbon degradation, the transcription of GHs remained stable across all treatments (*p* > 0.05, Figure 2b); conversely, CEs, which mediate polysaccharide modification, were significantly upregulated under the N10 and N15 treatments (*p* < 0.05, Figure 2b). Notably, the ratio of carbon fixation to degradation transcriptional activity reached its maximum under the N10 treatment but drastically dropped below the control baseline under the N15 and N20 treatments (*p* < 0.05, Figure 2c).

Mantel tests revealed that transcriptional variations in carbon fixation and degradation pathways were significantly driven by soil physicochemical properties. Transcriptional shifts in fixation pathways, including the *rTCA* cycle, WL pathway, and 3-HP bicycle, were significantly and positively correlated with SOC, TN, MBN, and POC (*p* < 0.05, Figure 2d); among these, the *rTCA* cycle additionally exhibited significant positive correlations with MBC and MAOC. Within the degradation pathways, GHs displayed weak associations with environmental factors, whereas CEs were significantly and positively correlated with SOC and POC. Notably, the lignin-degrading AAs exhibited the most extensive environmental associations, being highly significantly correlated with SOC, TN, MBN, MBC, and POC (*p* < 0.05).

The dominant microbial taxa transcriptionally associated with these carbon turnover pathways exhibited distinct compositional divergence at both the phylum and genus levels (Figure S1). For carbon fixation, the phylum Chloroflexota accounted for the highest known transcriptional contributions to the CBB cycle, 4-HB cycle, and WL pathway (Figure S1a). In contrast, the taxa expressing transcripts related to the *rTCA* cycle were more evenly distributed, predominantly comprising the phyla Actinomycetota, Acidobacteriota, and Chloroflexota. At the genus level, *Sulfobacillus*, *Thermogemmatispora*, and *Tengunoibacter* were identified as the core genera exhibiting the highest transcriptional potential for the CBB, 4-HB, and WL pathways, respectively, whereas *Thermomonospora* showed the highest transcript abundance associated with the *rTCA* cycle (Figure S1b).

Regarding carbon degradation, the phylum Chloroflexota, specifically the genus *Thermogemmatispora*, emerged as the primary contributor to lignin degradation (AAs) (Figure S1). Polysaccharide modification (CEs) and pectin cleavage (PLs) were predominantly driven by the phylum Actinomycetota, with *Catenulispora*, *Bradyrhizobium*, and *Streptomyces* acting as the core functioning genera. Furthermore, the phyla Acidobacteriota and Chloroflexota were the foundational taxa for cellulose degradation (GHs), with *Dictyobacter* functioning as the dominant known genus (Figure S1b).

### 3.3. Structural Dynamics of the Rhizosphere Metabolome and Response Profiles of Core Differential Metabolites Under N Addition

**Figure 3:**
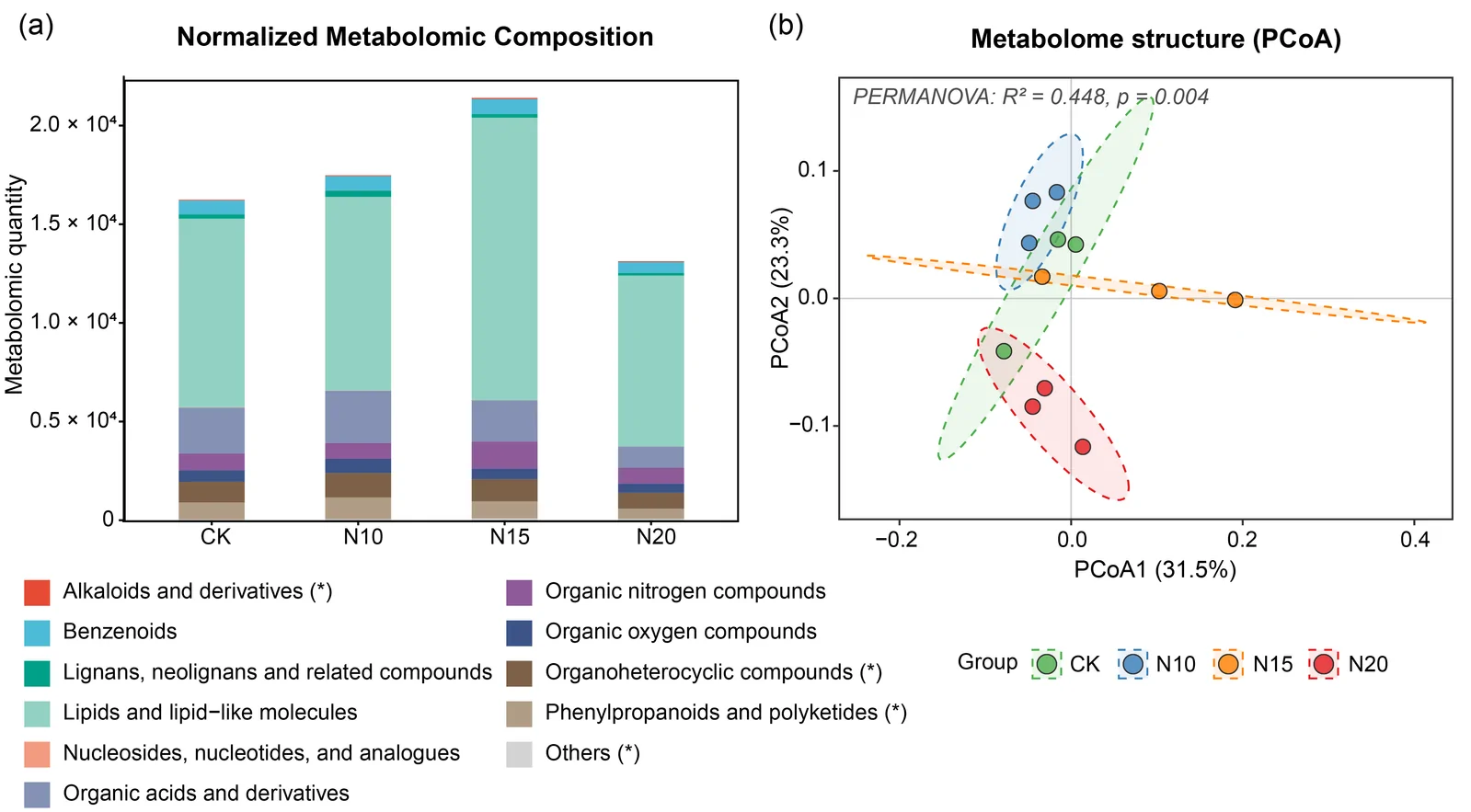
Rhizosphere metabolome responses to continuous six-year nitrogen (N) addition. (**a**) Normalized composition of major metabolic superclasses. The *y*-axis (metabolomic quantity) denotes the normalized total peak intensity. Asterisks (*) in the legend denote statistically significant differences among N treatments based on one-way ANOVA (*p* < 0.05). (**b**) Principal coordinate analysis (PCoA) of the overall metabolome structure. Dashed ellipses represent 95% confidence intervals. Compositional dissimilarities were statistically evaluated via permutational multivariate analysis of variance (PERMANOVA).

The compositional profiles and overall structure of rhizosphere metabolites shifted significantly along the continuous N addition gradient. Regarding chemical composition, lipids and lipid-like molecules exhibited absolute dominance across all treatments. Although the normalized total metabolite abundance displayed a non-significant nonlinear trend—peaking in the N15 treatment before declining under the N20 condition—no statistically significant differences were observed at the global level (Figure 3a). However, within the secondary metabolite superclasses, the abundances of alkaloids and derivatives, organoheterocyclic compounds, and phenylpropanoids and polyketides were significantly altered (*p* < 0.05). Furthermore, PCoA revealed that continuous N input significantly drove the overall compositional divergence of the rhizosphere metabolome (*R*^2^ = 0.448, *p* = 0.004, Figure 3b).

Utilizing PLS-DA, 33 significantly differential metabolites were robustly identified (VIP > 1 and FDR < 0.05, Figure S2). PCA based on these differential metabolic superclasses elucidated the primary variables (*R*^2^ = 0.874, *p* = 0.001, Figure S3). Based on the PCA loading scores, the N10 cluster was strongly associated with lipids and lipid-like molecules and organic acids and derivatives; the N15 cluster exhibited a high correlation with organic oxygen compounds and phenylpropanoids and polyketides (*p* < 0.001); whereas under the N20 treatment, organoheterocyclic compounds contributed substantially to the separation of the metabolome (*p* < 0.001).

**Figure 4:**
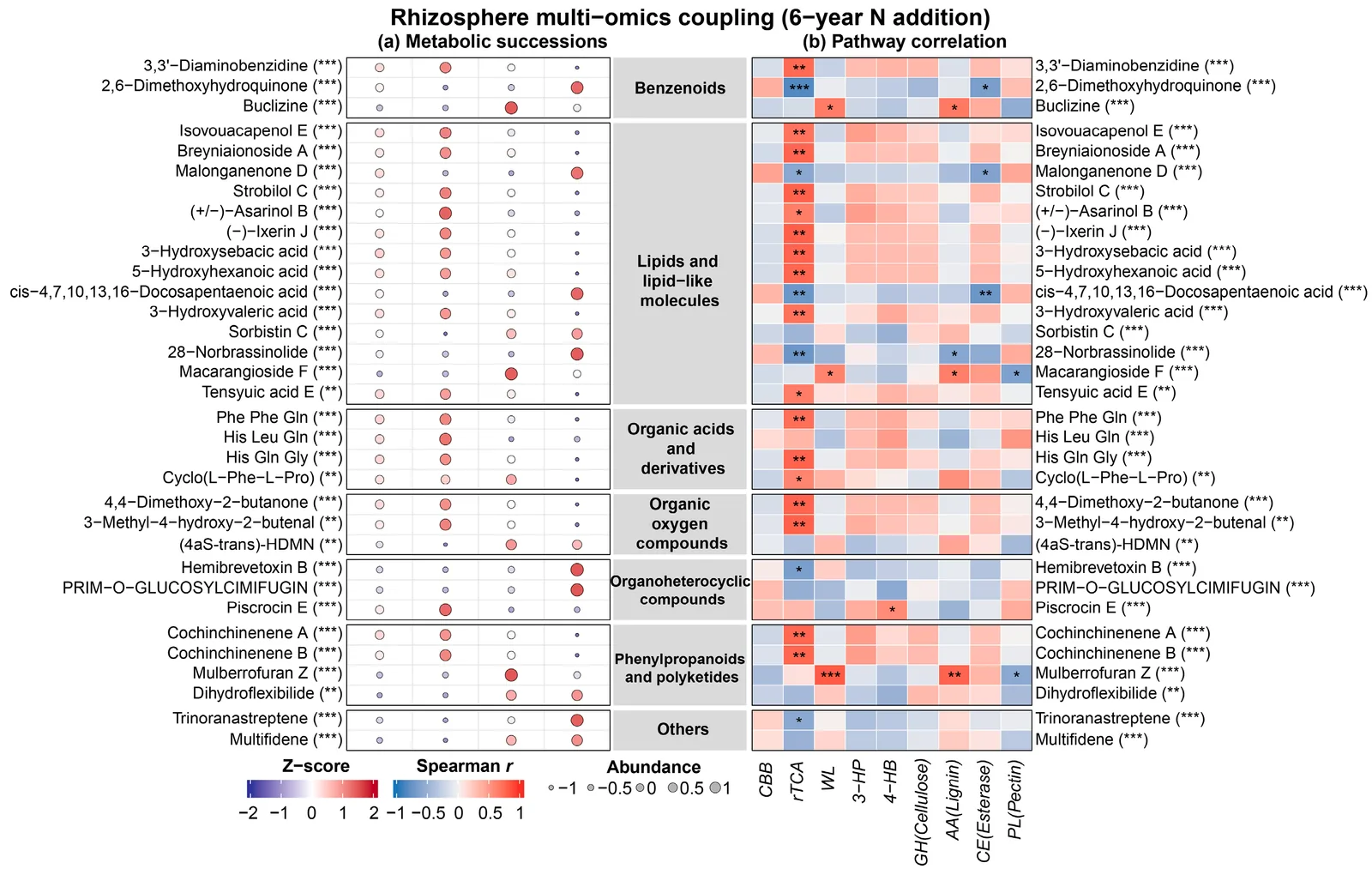
Coupled multi-omics analysis of rhizosphere carbon turnover under continuous six-year nitrogen (N) addition. (**a**) Response patterns of significantly differential metabolites. Bubble color intensity and size correspond to standardized Z-scores and relative abundances, respectively. Metabolites are systematically grouped according to their predefined metabolic superclasses. Asterisks adjacent to metabolite names denote statistically significant abundance variations across the N input gradient based on one-way analysis of variance (ANOVA). (**b**) Spearman correlation heatmap linking the differential metabolites to the transcriptional activities of core carbon-cycling pathways. Red and blue cells denote positive and negative correlation coefficients (*r*), respectively. Asterisks within the matrix cells indicate statistical significance levels (* *p* < 0.05, ** *p* < 0.01, *** *p* < 0.001).

The standardized relative abundances (Z-scores) of these core differential metabolites exhibited highly heterogeneous response trajectories (Figure 4a). Under the N10 conditions, the majority of organic acids and derivatives (e.g., Phe-Phe-Gln and His-Gln-Gly), specific lipid molecules (e.g., isovouacapenol E and breyniaionoside A), and phenylpropanoids and polyketides (e.g., cochinchinenene A) were significantly enriched, reaching their peak abundances. Under the N15 treatment, the lipid macarangioside F, the benzenoid buclizine, and the phenylpropanoid mulberrofuran Z reached their maximum concentrations. Conversely, under the N20 treatment, the abundances of most aforementioned enriched metabolites rapidly collapsed. Concurrently, the relative abundances of specific compounds—such as malonganenone D, hemibrevetoxin B, 28-norbrassinolide, and *cis*-4,7,10,13,16-docosapentaenoic acid—experienced a significant surge.

### 3.4. Linking Rhizosphere Metabolic Profiles to Transcriptional Activity and Mechanistic Drivers of SOC Accumulation

Spearman’s correlation analysis demonstrated statistically significant associations between the relative abundances of differential metabolites and the transcriptional activities of specific carbon-cycling pathways (Figure 4b). Regarding carbon fixation, the transcriptional activity of the *rTCA* cycle displayed highly significant linear correlations (either positive or negative, *p* < 0.01) with most lipid molecules (e.g., *breyniaionoside A*), organic acids and derivatives (e.g., Phe-Phe-Gln, His-Gln-Gly), and phenylpropanoids; the WL pathway showed a significant positive correlation with the phenylpropanoid *mulberrofuran Z* (*p* < 0.001). Within the degradation pathways, the transcriptional activities of the lignin oxidation (AAs) and polysaccharide modification (CEs) pathways likewise exhibited significant correlations with specific lipids (e.g., *macarangioside F*, *cis*-4,7,10,13,16-docosapentaenoic acid) (*p* < 0.05).

**Figure 5:**
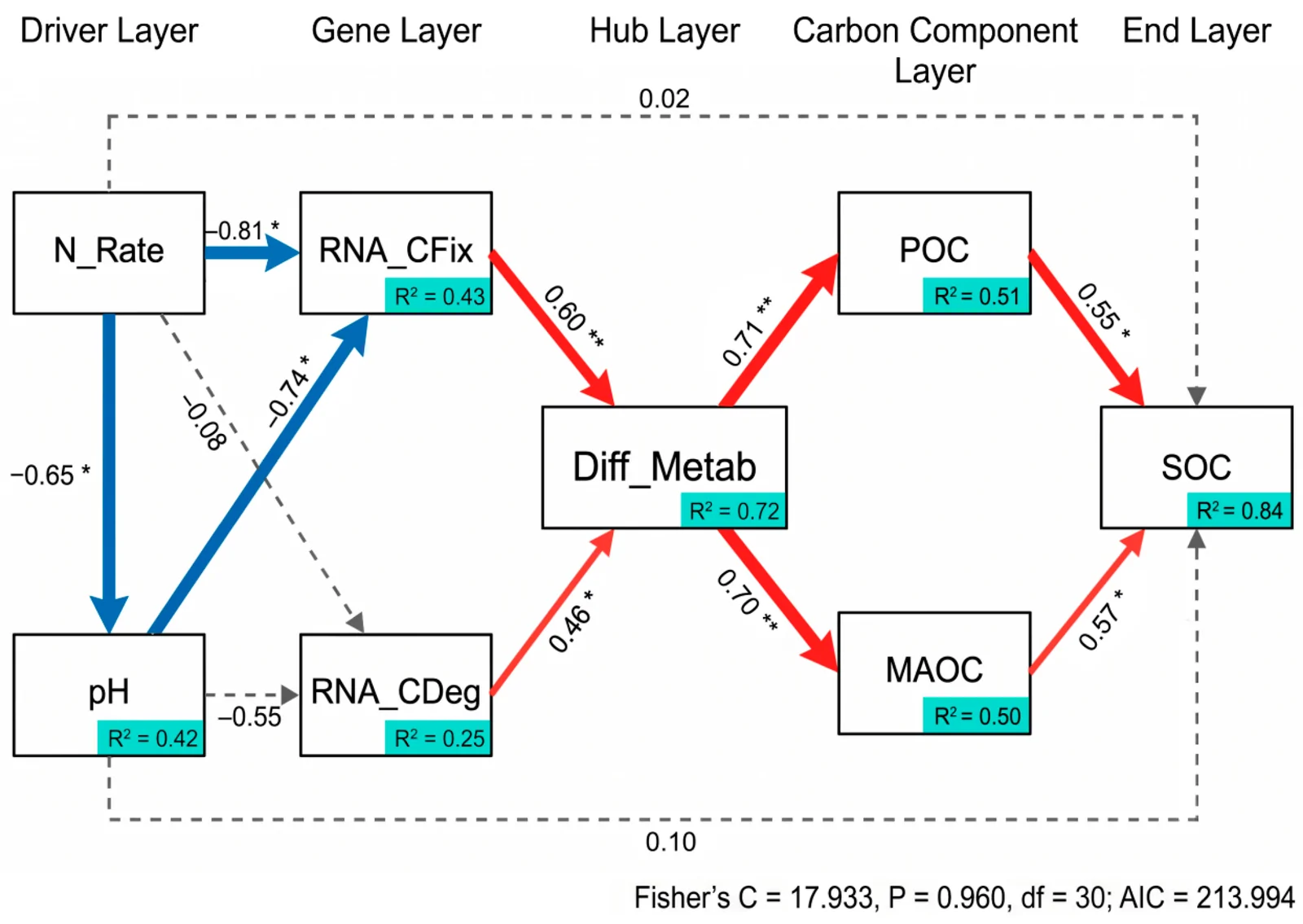
Piecewise structural equation model (pSEM) delineating the cascading effects of continuous six-year nitrogen addition on rhizosphere carbon metabolic succession and soil organic carbon (SOC). Red and blue solid arrows indicate significant positive and negative effects, respectively, with thickness proportional to standardized path coefficients. Gray dashed arrows represent non-significant pathways. Cyan highlights display the variance explained (*R*^2^) for endogenous variables. Asterisks indicate statistical significance (* *p* < 0.05, ** *p* < 0.01). Model abbreviations: N_Rate, nitrogen addition gradient; RNA_CFix, transcriptional activity of carbon fixation genes; RNA_CDeg, transcriptional activity of carbon degradation genes; Diff_Metab, variance of core differential metabolites; POC, particulate organic carbon; MAOC, mineral-associated organic carbon; SOC, total soil organic carbon.

The pSEM further resolved the cascading pathways through which environmental variables, gene transcriptional activity, and differential metabolites collectively dictate soil carbon fractions (Fisher’s *C* = 17.933, *p* = 0.960; Figure 5). Path analysis revealed that N addition exerted a significant negative effect on soil pH (*β* = −0.65). Both N addition and soil pH negatively regulated the transcriptional activity of carbon fixation genes (RNA_CFix) (*β* = −0.81 and −0.74, respectively). Furthermore, the transcriptional activities of both carbon fixation and degradation (RNA_CDeg) genes exhibited significant positive effects on the variance in differential metabolite profiles (Diff_Metab) (*β* = 0.60 and 0.46, respectively). Notably, these differential metabolites exerted highly significant positive effects on both POC and MAOC (*β* = 0.71 and 0.70, *p* < 0.01), which in turn significantly promoted the total SOC content (*β* = 0.55 and 0.57, *p* < 0.05).

## 4. Discussion

### 4.1. Regulatory Mechanisms of Continuous N Addition on the Rhizosphere Microenvironment and Carbon-Cycling Transcriptional Activity

Following continuous six-year N addition, total SOC and its physical fractions exhibited pronounced nonlinear responses. Consistent with global meta-analyses [56,57], the N10 input alleviated systemic N limitation, significantly enriching SOC, POC, and MAOC [58]. Our physicochemical data corroborate this dynamic: under the N10 treatment, rhizosphere MBN increased significantly relative to the unamended control (CK), alongside a parallel enrichment in DON. This dynamic provides fundamental substrates for microbial assimilation, cellular growth, and biomass accumulation. Based on our 6-year continuous observations, once the N input exceeded 10 kg N ha^−1^ yr^−1^ (i.e., sustained inputs at N15 and N20), the treatments triggered severe soil acidification, effectively constraining the carbon sequestration capacity of the microhabitat and precipitating a rapid collapse in SOC and its associated fractions [4,59]. These findings align with recent field observations in alpine meadows across the Qinghai–Tibet Plateau [60]. Furthermore, while short-term experiments (<3 years) frequently report SOC accumulation due to initial nutrient alleviation, and decadal studies (>10 years) often document severe carbon loss, this 6-year study provides insights into the intermediate transitional phase. It illustrates the process by which continuous N addition alters soil physicochemical properties, shifting the microbial community from a carbon-assimilating state to a degradation-dominated state. The data underscore that sustained N enrichment exceeding this threshold not only exacerbates soil acidification but irreparably disrupts the stoichiometric equilibrium of the system [61]. Consequently, the carbon sink function of alpine grassland ecosystems remains highly vulnerable to exogenous nutrient perturbations.

In response to environmental perturbations, soil microbiomes rapidly modulate carbon turnover dynamics by reprogramming the transcription of core functional genes [62]. Leveraging metatranscriptomic profiling, we demonstrated that the transcriptional trajectories of rhizosphere carbon turnover pathways shifted significantly across the N addition gradient. Under the relatively nutrient-replete conditions of the N10 threshold, the transcription of carbon fixation pathways, particularly the *rTCA* cycle, was significantly upregulated. This drove a concomitant increase in the transcriptional ratio of carbon fixation to degradation, which flawlessly mirrors our observed trends of MBN enrichment and SOC accumulation, corroborating recent findings [63]. However, under the N15 and N20 treatments, pSEM revealed that severe soil acidification exerted robust negative feedback on carbon-fixing transcription. Consequently, the fixation-to-degradation transcriptional ratio plummeted, and the carbon turnover equilibrium shifted toward a degradation-dominated regime [64]. Furthermore, these transcriptional variations exhibited significant positive correlations with critical environmental factors (e.g., SOC and TN), substantiating the prevailing consensus that microbiomes execute transcriptional resource reallocation under severe environmental stress [65,66]. Under conditions of acid stress and stoichiometric imbalance, thermodynamically costly carbon fixation pathways are suppressed, whereas carbon degradation pathways maintain high baseline activity. This suggests a survival strategy wherein microbes preferentially allocate energy toward basal metabolic maintenance rather than carbon assimilation [67,68]. Notably, unlike other highly volatile pathways, the transcriptional activity of GHs remained remarkably stable across all treatments. This resilience likely stems from the extensive biochemical functional redundancy of GHs distributed across diverse microbial lineages [69], buffering their immediate transcriptional responses to environmental disturbances.

Furthermore, our taxonomic contribution analysis revealed that the transcriptional vigor of these key carbon turnover pathways was not homogeneously driven by the bulk microbiome; rather, it was exclusively dictated by the metabolic responses of core functional taxa [70]. The phylum Chloroflexota emerged as a dominant contributor to core carbon fixation pathways—such as the CBB cycle and the WL pathway—as well as to degradation networks. This robust transcriptional footprint highlights the exceptional adaptive capacity of quintessential oligotrophic taxa within harsh, cold, and nutrient-deprived alpine habitats [70,71]. At the genus level, *Tengunoibacter*, *Dictyobacter*, and *Thermogemmatispora* functioned as the central keystone drivers within their respective specific pathways. The functional relevance of these specific taxa is strongly supported by empirical evidence from other terrestrial ecosystems [72]. For instance, species within *Sulfobacillus* and *Thermogemmatispora* have been widely validated as versatile carbon cyclers in various soil and extreme acidic environments, possessing robust genomic repertoires for both autotrophic carbon assimilation and recalcitrant organic matter degradation [73,74]. These multi-omics insights indicate that exogenous N inputs fundamentally reshape the rhizosphere microenvironment, thereby selectively filtering and stimulating specific microbial taxa equipped with indispensable functional traits. Ultimately, this targeted taxonomic filtering drives the critical carbon cycling processes underlying the alpine meadow microecosystem [75,76].

### 4.2. The Mediating Role of Differential Metabolites in Rhizosphere Soil Organic Carbon Turnover

Rhizosphere metabolite profiles are directly shaped by microbial transcriptional activity. Furthermore, these metabolites serve as essential precursors that drive the differentiation of the SOC pool [77]. According to microbial resource allocation theory [78], under varying degrees of environmental stress, microbial energy allocation necessitates a trade-off among assimilatory growth, basal maintenance, and stress responses [79,80]. Our findings robustly corroborate this theoretical framework. Under the N10 treatment, the enrichment of rhizosphere MBN and DON indicated the alleviation of systemic N limitation, paralleled by a significant enhancement in the transcriptional activity of the *rTCA* cycle. Concurrently, the relative abundances of characteristic organic acids and derivatives (e.g., Phe-Phe-Gln and His-Gln-Gly) and specific lipids (e.g., isovouacapenol E and breyniaionoside A) were significantly upregulated, reaching their peak concentrations. This sequential cascade—from the amelioration of the physicochemical microhabitat to the activation of specific transcriptional pathways and the subsequent accumulation of primary metabolites—suggests that the N10 input shifts microbial resource allocation toward anabolic growth [12,65,71]. Consequently, assimilated carbon is preferentially channeled into primary metabolism and cellular biomass synthesis [81]. Simultaneously, these microbe-derived macromolecules (lipids and organic acids) provide essential fundamental precursors for the stabilization of soil organic matter [77].

Under the N15 treatment, specific lipids (e.g., macarangioside F), benzenoids, and phenylpropanoids and polyketides (e.g., mulberrofuran Z) reached their abundance maxima. This indicates a fundamental shift in the accumulation patterns of rhizosphere metabolites as the N input transitioned from alleviating nutrient limitation to inducing environmental stress—a phenomenon entirely consistent with established theories of N threshold effects and metabolic reprogramming [16,82]. Under the N10 loading, the enriched relative abundances of metabolites such as lipids and organic acids supplied a critical metabolic foundation for carbon pool accretion [81]. In contrast, under conditions where N inputs > 10 kg N ha^−1^ yr^−1^ (N15 and N20), the transcription of carbon-fixing genes was strongly suppressed, while carbon-degrading transcription was relatively sustained. This transcriptional decoupling, coupled with the downregulated activity of carbon-assimilating metabolic networks, constitutes the intrinsic mechanistic basis for the observed collapse in SOC fractions. This dynamic underscores how environmental stress compels microbiomes to recalibrate their survival strategies [78].

When N inputs escalated to the N20 level, persistent soil acidification imposed intense selective stress on the rhizosphere microhabitat [13,65], driving further deviations in microbial transcriptional profiles. Under such acid stress, the microbiome drastically downregulated the transcription of carbon-fixing pathways; however, the expression of carbon-degrading genes, specifically those governing lignin oxidation, remained robustly maintained [83]. Correspondingly, the relative abundances of primary lipids and organic acids plummeted within the N20 treatment, whereas specific Organoheterocyclic compounds and polyunsaturated fatty acids (e.g., *cis*-4,7,10,13,16-docosapentaenoic acid) exhibited significant enrichment. Consistent with the existing literature, this reduction in primary metabolites alongside the accumulation of specific stress-associated compounds exemplifies a classic microbial “growth–maintenance trade-off” [84,85]. Confronted with severe resource and environmental constraints, the microbiome strategically reroutes energy fluxes away from anabolic growth and firmly toward stress response and basal maintenance strategies.

### 4.3. Synergistic Responses of Physical SOC Fractions Mediated by Rhizosphere Transcription and Metabolism

The transcriptional and metabolic activities of rhizosphere microbiomes fundamentally drive SOC dynamics in alpine grasslands. The pSEM developed in this study quantitatively resolved the cascading pathways through which environmental shifts impact carbon pool stability via these micro-ecological processes. Our results indicate that continuous N addition triggered soil acidification, which subsequently exerted a negative feedback effect on carbon-fixing transcription. This divergent transcriptional response between carbon fixation and degradation likely mediated the profound compositional shifts observed in the metabolome. Ultimately, these differential metabolites were tightly linked to the subsequent accumulation of POC and MAOC, thereby dictating the overall trajectory of SOC storage [8,27].

These findings strongly align with the MCP conceptual framework [29,81], which posits that microbe-derived metabolites and necromass serve as essential precursors for the formation of stable mineral-associated organic carbon [86]. Recent studies further emphasize that these microbial ex vivo compounds are preferentially stabilized within the MAOC pool due to their high affinity for mineral surfaces [87]. Our integrated multi-omics results corroborate this theory at the transcriptional–metabolic nexus: under the N10 input, enhanced *rTCA* cycle transcription occurred synchronously with enriched abundances of specific lipids and organic acids. These primary metabolites likely function as fundamental precursors, providing the material basis for POC and MAOC accretion. Conversely, under the N15 and N20 inputs, the proportional upregulation of degradation-related transcripts significantly depleted these carbonaceous precursors. This shift in substrate availability fundamentally restricted the effective flux and stabilization of carbon into stable physical pools [86].

The path analysis further elucidated the synergistic roles of POC and MAOC in driving total SOC accumulation. Structural equation pathways demonstrated that N-induced soil acidification and transcriptional suppression initially depleted key carbon-containing metabolites; this subsequently triggered a collapse in POC and MAOC contents, ultimately halting further SOC sequestration [87]. While several short-term controlled experiments suggest that exogenous N addition can promote carbon sequestration by stimulating plant productivity [57,88], our findings reveal that continuous six-year N inputs exceeding 10 kg N ha^−1^ yr^−1^ precipitate severe soil acidification. This, in turn, fundamentally alters the microscopic synergistic responses of rhizosphere transcription and metabolism. Such molecular-level metabolic decoupling effectively neutralizes any short-term carbon accrual achieved via nutrient supplementation [65,68]. Therefore, accurately forecasting carbon pool dynamics in alpine grassland ecosystems under continuous N deposition mandates the comprehensive integration of microbial transcriptional and metabolic profiling.

It must be objectively acknowledged that the pSEM results presented herein represent statistical associations rather than strict empirical causality. Such statistical modeling serves as a powerful framework to generate mechanistic hypotheses, but it cannot replace direct experimental validation of these causal relationships. Furthermore, while our taxonomic inferences robustly identify the core contributing genera at the transcriptional level, we explicitly acknowledge that owing to complex post-transcriptional regulations and enzyme kinetics, RNA transcript abundance does not strictly equate to in vivo protein activity or actual functional metabolic fluxes [40,69]. Consequently, the extent to which these specific taxa ultimately drive physical carbon fluxes requires further empirical validation. Future investigations should incorporate stable isotope probing (SIP) or targeted metaproteomics to precisely quantify the definitive carbon sequestration contributions of these core pathways. Furthermore, different plant species vary in their nitrogen absorption capacities. This difference can alter the actual amount of nitrogen involved in soil processes. Therefore, investigating species-specific plant–microbe interactions will be a crucial step for future research.

## 5. Conclusions

Continuous six-year exogenous N addition exerts a pronounced nonlinear threshold effect on rhizosphere carbon sequestration in Qinghai–Tibet Plateau alpine grasslands. The N10 input (10 kg N ha^−1^ yr^−1^) stimulates the transcription of core carbon fixation pathways. However, as N loading surpasses this threshold, the microbial transcriptional equilibrium fundamentally shifts toward a degradation-dominated regime. Concurrently, the rhizosphere metabolome undergoes a significant compositional evolution: while the N10 treatment promotes the accumulation of primary precursor metabolites (e.g., lipids and organic acids), the N15 and N20 inputs drive the accelerated decomposition of available carbon substrates. This metabolic shift critically restricts the synthesis of fundamental precursors required for the maintenance of the SOC pool. Ultimately, the severe soil physicochemical alterations induced by excessive N addition, coupled with the resultant transcriptional decoupling and metabolic reprogramming, collectively undermine the global stability of the SOC reservoir. Therefore, against the backdrop of escalating global atmospheric N deposition, this study highlights the severe ecological risks that sustained N enrichment (>10 kg N ha^−1^ yr^−1^) poses to the carbon sink function of fragile alpine ecosystems. Acknowledging the spatial heterogeneity of alpine environments and the constraints of current sample sizes, future research must integrate stable isotope probing (SIP) and multi-site, longitudinal in situ observations to precisely quantify and validate the empirical carbon sequestration contributions of these micro-scale turnover pathways.

## Data Availability

The original contributions presented in the study are included in the article/Supplementary Materials, further inquiries can be directed to the corresponding author.

## Supplementary Materials

The following supporting information can be downloaded at https://www.mdpi.com/article/10.3390/microorganisms14081808/s1. Figure S1. Multilevel taxonomic contributions to carbon fixation and degradation pathways. Figure S2. Identification of differential metabolites via partial least squares-discriminant analysis (PLS-DA). Figure S3. Principal component analysis (PCA) of rhizosphere differential metabolites categorized by metabolic superclasses.

## Abbreviations

The following abbreviations are used in this manuscript:

N	Nitrogen
SOC	Soil Organic Carbon
POC	Particulate Organic Carbon
MAOC	Mineral-Associated Organic Carbon
CK	Control
MBC	Microbial Biomass Carbon
MBN	Microbial Biomass Nitrogen
TN	Total Nitrogen
DOC	Dissolved Organic Carbon
DON	Dissolved Organic Nitrogen
rTCA	Reductive Tricarboxylic Acid
GH	Glycoside Hydrolase
CE	Carbohydrate Esterase
AA	Auxiliary Activities
pSEM	Piecewise Structural Equation Modeling
PCA	Principal Component Analysis
PCoA	Principal Coordinate Analysis
PLS-DA	Partial Least Squares–Discriminant Analysis
PL	Polysaccharide Lyase
CBB	Calvin–Benson–Bassham
WL	Wood–Ljungdahl
3-HP	3-Hydroxypropionate
4-HB	4-Hydroxybutyrate
CUE	Carbon Use Efficiency
CAZy	Carbohydrate-Active enZymes
KEGG	Kyoto Encyclopedia of Genes and Genomes

## References

[1] Galloway J.N., Bleeker A., Erisman J.W. (2021). The human creation and use of reactive nitrogen: A global and regional perspective. Annu. Rev. Environ. Resour..

[2] Legg S. (2021). IPCC, 2021: Climate change 2021-the physical science basis. Interaction.

[3] Friedlingstein P., O’Sullivan M., Jones M.W., Andrew R.M., Bakker D.C., Hauck J., Landschützer P., Le Quéré C., Li H., Luijkx I.T. (2025). Global carbon budget 2025. Earth Syst. Sci. Data Discuss..

[4] Zhou H., Yang X., Zhou C., Shao X., Shi Z., Li H., Su H., Qin R., Chang T., Hu X. (2023). Alpine grassland degradation and its restoration in the Qinghai–Tibet plateau. Grasses.

[5] Huang W., Tong L., Wu Z., Hu M., Liu S., Ye Y., Han Y. (2026). Effect of Nitrogen on Interaction Between Carbon, Nitrogen and Phosphorus Cycles in High-Altitude Apple Orchards. Agriculture.

[6] Stockmann U., Adams M.A., Crawford J.W., Field D.J., Henakaarchchi N., Jenkins M., Minasny B., McBratney A.B., De Courcelles V.d.R., Singh K. (2013). The knowns, known unknowns and unknowns of sequestration of soil organic carbon. Agric. Ecosyst. Environ..

[7] Bai Y., Cotrufo M.F. (2022). Grassland soil carbon sequestration: Current understanding, challenges, and solutions. Science.

[8] Xu Y., Zhao Y., Cha X., Yang W., Zheng M., Liu S., Wang Y., Cai A., Han X., Yang G. (2024). Nitrogen addition-driven soil organic carbon stability depends on the fractions of particulate and mineral-associated organic carbon. Nutr. Cycl. Agroecosyst..

[9] Chen H., Ju P., Zhu Q., Xu X., Wu N., Gao Y., Feng X., Tian J., Niu S., Zhang Y. (2022). Carbon and nitrogen cycling on the Qinghai–Tibetan Plateau. Nat. Rev. Earth Environ..

[10] Ma W., Tang S., Dengzeng Z., Zhang D., Zhang T., Ma X. (2022). Root exudates contribute to belowground ecosystem hotspots: A review. Front. Microbiol..

[11] Hu J., Huang C., Zhou S., Kuzyakov Y. (2022). Nitrogen addition to soil affects microbial carbon use efficiency: Meta-analysis of similarities and differences in 13C and 18O approaches. Glob. Change Biol..

[12] Jiang Y., Su T., Wang H., Yang Q., Lu J., Fu Q., Mao H., Xu W., Luo Y., Liu W. (2024). Deep soil microbial carbon use efficiency responds stronger to nitrogen deposition than top soil in tropical forests, southern China. Plant Soil.

[13] Zhang L., Zhao Z., Jiang B., Baoyin B., Cui Z., Wang H., Li Q., Cui J. (2024). Effects of long-term application of nitrogen fertilizer on soil acidification and biological properties in China: A meta-analysis. Microorganisms.

[14] He C., Ruan Y., Jia Z. (2024). Effects of nitrogen addition on soil microbial biomass: A meta-analysis. Agriculture.

[15] Xiong M., Jiang W., Zou S., Kang D., Yan X. (2023). Microbial carbohydrate-active enzymes influence soil carbon by regulating the of plant-and fungal-derived biomass decomposition in plateau peat wetlands under differing water conditions. Front. Microbiol..

[16] Pei Z., Shen Q., Shang X., Hong M. (2024). Different responses of lipids and lignin phenols to nitrogen addition in meadow grassland soil. J. Soils Sediments.

[17] Li K., Lin H., Han M., Yang L. (2024). Soil metagenomics reveals the effect of nitrogen on soil microbial communities and nitrogen-cycle functional genes in the rhizosphere of *Panax ginseng*. Front. Plant Sci..

[18] Kuzyakov Y., Blagodatskaya E. (2015). Microbial hotspots and hot moments in soil: Concept & review. Soil Biol. Biochem..

[19] Sokol N.W., Kuebbing S.E., Karlsen-Ayala E., Bradford M.A. (2019). Evidence for the primacy of living root inputs, not root or shoot litter, in forming soil organic carbon. New Phytol..

[20] Rahul R., Sharma P., Singh A., Singh J., Kumar M. (2022). Soil enzymes and their role in soil health improvement. Advances in Agricultural and Industrial Microbiology: Volume 1: Microbial Diversity and Application in Agroindustry.

[21] Cheng B., Liu H., Bai J., Li J. (2022). Soil fungal composition drives ecosystem multifunctionality after long-term field nitrogen and phosphorus addition in alpine meadows on the Tibetan plateau. Plants.

[22] Shi G., Zhang Z., Ma L., Liu Y., Wang Y., Uwamungu J.Y., Feng H., Dong S., Yao B., Zhou H. (2024). Nitrogen addition drives changes in arbuscular mycorrhizal fungal richness through changes in plant species richness in revegetated alpine grassland. Fungal Ecol..

[23] Liao J., Dou Y., Yang X., An S. (2023). Soil microbial community and their functional genes during grassland restoration. J. Environ. Manag..

[24] Yang C., Zhang H., Zhao X., Liu P., Wang L., Wang W. (2023). A functional metagenomics study of soil carbon and nitrogen degradation networks and limiting factors on the Tibetan plateau. Front. Microbiol..

[25] Yang J., Lee J., Choi J., Ma L., Heaton E.A., Howe A. (2022). Response of Total (DNA) and metabolically active (RNA) microbial communities in *Miscanthus *× *Giganteus* cultivated soil to different nitrogen fertilization rates. Microbiol. Spectr..

[26] Dai S., Wu H., Chen H., Wang Z., Yu X., Wang L., Jia X., Qin C., Zhu Y., Yi K. (2023). Comparative transcriptome analyses under individual and combined nutrient starvations provide insights into N/P/K interactions in rice. Plant Physiol. Biochem..

[27] Rosado-Porto D., Ratering S., Moser G., Deppe M., Müller C., Schnell S. (2022). Soil metatranscriptome demonstrates a shift in C, N, and S metabolisms of a grassland ecosystem in response to elevated atmospheric CO_2_. Front. Microbiol..

[28] Sokol N.W., Bradford M.A. (2019). Microbial formation of stable soil carbon is more efficient from belowground than aboveground input. Nat. Geosci..

[29] Zhu X., Jackson R.D., DeLucia E.H., Tiedje J.M., Liang C. (2020). The soil microbial carbon pump: From conceptual insights to empirical assessments. Glob. Change Biol..

[30] Liang C., Amelung W., Lehmann J., Kästner M. (2019). Quantitative assessment of microbial necromass contribution to soil organic matter. Glob. Change Biol..

[31] Wang Y.-C., Lv Y.-H., Yang W.-X., Wang C. (2024). Revisiting the linkage between engineered ecosystem function and the microbial community: A review from ecological perspectives. Crit. Rev. Environ. Sci. Technol..

[32] Blazewicz S.J., Barnard R.L., Daly R.A., Firestone M.K. (2013). Evaluating rRNA as an indicator of microbial activity in environmental communities: Limitations and uses. ISME J..

[33] Wang W., Liu X., Guan L. (2024). High atmospheric dissolved organic nitrogen deposition in southeast Tibet. Heliyon.

[34] Wu Z., Han Y., Huang W., Hu M., Tong L., Zhang S., Liu S., Ye Y. (2026). 6-year nitrogen addition alters fungal network structure and extracellular enzyme activities in alpine grasslands: Divergent responses between rhizosphere and bulk soils. Chem. Biol. Technol. Agric..

[35] Kuzyakov Y., Razavi B.S. (2019). Rhizosphere size and shape: Temporal dynamics and spatial stationarity. Soil Biol. Biochem..

[36] Chen H., Ma K., Lu C., Fu Q., Qiu Y., Zhao J., Huang Y., Yang Y., Schadt C.W., Chen H. (2022). Functional redundancy in soil microbial community based on metagenomics across the globe. Front. Microbiol..

[37] Jones D.L., Willett V.B. (2006). Experimental evaluation of methods to quantify dissolved organic nitrogen (DON) and dissolved organic carbon (DOC) in soil. Soil Biol. Biochem..

[38] Wilke B.-M. (2005). Determination of chemical and physical soil properties. Monitoring and Assessing Soil Bioremediation.

[39] Joergensen R.G. (1996). The fumigation-extraction method to estimate soil microbial biomass: Calibration of the kEC value. Soil Biol. Biochem..

[40] Dahl M.B., Brachmann S., Söllinger A., Schnell M., Ahlers L., Wutkowska M., Hoff K.J., Nath N., Groß V., Wang H. (2025). Quantifying soil microbiome abundance by metatranscriptomics and complementary molecular techniques—Cross-validation and perspectives. Mol. Ecol. Resour..

[41] Schmieder R., Edwards R. (2011). Quality control and preprocessing of metagenomic datasets. Bioinformatics.

[42] Langmead B., Salzberg S.L. (2012). Fast gapped-read alignment with Bowtie 2. Nat. Methods.

[43] Zhao S., Ye Z., Stanton R. (2020). Misuse of RPKM or TPM normalization when comparing across samples and sequencing protocols. Rna.

[44] Cantalapiedra C.P., Ana H.-P., Ivica L., Peer B., Jaime H.C. (2021). eggNOG-mapper v2: Functional Annotation, Orthology Assignments, and Domain Prediction at the Metagenomic Scale. Mol. Biol. Evol..

[45] Kanehisa M., Furumichi M., Sato Y., Kawashima M., Ishiguro-Watanabe M. (2023). KEGG for taxonomy-based analysis of pathways and genomes. Nucleic Acids Res..

[46] Drula E., Garron M.-L., Dogan S., Lombard V., Henrissat B., Terrapon N. (2022). The carbohydrate-active enzyme database: Functions and literature. Nucleic Acids Res..

[47] Berg I.A. (2011). Ecological aspects of the distribution of different autotrophic CO2 fixation pathways. Appl. Environ. Microbiol..

[48] Smith C.A., Want E.J., O’Maille G., Abagyan R., Siuzdak G. (2006). XCMS: Processing mass spectrometry data for metabolite profiling using nonlinear peak alignment, matching, and identification. Anal. Chem..

[49] Dunn W.B., Broadhurst D., Begley P., Zelena E., Francis-McIntyre S., Anderson N., Brown M., Knowles J.D., Halsall A., Haselden J.N. (2011). Procedures for large-scale metabolic profiling of serum and plasma using gas chromatography and liquid chromatography coupled to mass spectrometry. Nat. Protoc..

[50] Want E.J., Masson P., Michopoulos F., Wilson I.D., Theodoridis G., Plumb R.S., Shockcor J., Loftus N., Holmes E., Nicholson J.K. (2013). Global metabolic profiling of animal and human tissues via UPLC-MS. Nat. Protoc..

[51] Djoumbou Feunang Y., Eisner R., Knox C., Chepelev L., Hastings J., Owen G., Fahy E., Steinbeck C., Subramanian S., Bolton E. (2016). ClassyFire: Automated chemical classification with a comprehensive, computable taxonomy. J. Cheminform..

[52] R Core Team (2020). RA Language and Environment for Statistical Computing.

[53] Wickham H. (2016). Data analysis. ggplot2: Elegant Graphics for Data Analysis.

[54] Lefcheck J.S. (2016). piecewiseSEM: Piecewise structural equation modelling in r for ecology, evolution, and systematics. Methods Ecol. Evol..

[55] Shipley B. (2009). Confirmatory path analysis in a generalized multilevel context. Ecology.

[56] Ye Y., Wu Z., Zhang S., Tong L., Huang W., Cui Z., Han Y. (2025). Effects of nitrogen addition on SOC in alpine grasslands of the Qinghai-Tibetan Plateau and adjacent mountain regions: A meta-analysis. Front. Environ. Sci..

[57] Yang X., Ma S., Huang E., Zhang D., Chen G., Zhu J., Ji C., Zhu B., Liu L., Fang J. (2025). Nitrogen addition promotes soil carbon accumulation globally. Sci. China Life Sci..

[58] Tang S., Pan W., Yang Y., Luo Z., Wanek W., Kuzyakov Y., Marsden K.A., Liang G., Chadwick D.R., Gregory A.S. (2025). Soil carbon sequestration enhanced by long-term nitrogen and phosphorus fertilization. Nat. Geosci..

[59] Luo M., Moorhead D.L., Ochoa-Hueso R., Mueller C.W., Ying S.C., Chen J. (2022). Nitrogen loading enhances phosphorus limitation in terrestrial ecosystems with implications for soil carbon cycling. Funct. Ecol..

[60] Xu C., Xu X., Ju C., Chen H.Y., Wilsey B.J., Luo Y., Fan W. (2021). Long-term, amplified responses of soil organic carbon to nitrogen addition worldwide. Glob. Change Biol..

[61] Xing W., Lu X., Geng S., Ding J., Bai Y. (2023). Mechanisms underlying the negative effects of nitrogen addition on soil nematode communities in global grassland ecosystems. Geoderma.

[62] Neff J.C., Townsend A.R., Gleixner G., Lehman S.J., Turnbull J., Bowman W.D. (2002). Variable effects of nitrogen additions on the stability and turnover of soil carbon. Nature.

[63] Cao X., Wang J., Bahethan B., Chen Y., Liu J., Lü G. (2025). Microbial Communities and Environmental Factors Interact to Regulate Soil Respiration Under Nitrogen Addition Conditions in Alpine Meadows in Northwest China. Microorganisms.

[64] Liu Y., Dou X., Wang C., Song M., Liu D., Liu X., Zhu Q. (2025). Long-term nitrogen addition enhanced soil carbon sequestration through coupled physicochemical and microbial mechanisms in a temperate forest. J. Environ. Manag..

[65] Xing W., Lu X., Ying J., Lan Z., Chen D., Bai Y. (2022). Disentangling the effects of nitrogen availability and soil acidification on microbial taxa and soil carbon dynamics in natural grasslands. Soil Biol. Biochem..

[66] Patoine G., Eisenhauer N., Cesarz S., Phillips H.R., Xu X., Zhang L., Guerra C.A. (2022). Drivers and trends of global soil microbial carbon over two decades. Nat. Commun..

[67] Liu H.Y., Huang N., Zhao C.M., Li J.H. (2023). Responses of carbon cycling and soil organic carbon content to nitrogen addition in grasslands globally. Soil Biol. Biochem..

[68] Li S., Tang S., Ju X., Zhu Z., Zhang Y., Chen H., Jin K. (2024). Soil acidification drives the negative effects of nitrogen enrichment on soil microbial biomass at the global scale. Plant Soil.

[69] Berlemont R., Martiny A.C. (2013). Phylogenetic distribution of potential cellulases in bacteria. Appl. Environ. Microbiol..

[70] Lin X., Tfaily M.M., Green S.J., Steinweg J.M., Chanton P., Imvittaya A., Chanton J.P., Cooper W., Schadt C., Kostka J.E. (2014). Microbial metabolic potential for carbon degradation and nutrient (nitrogen and phosphorus) acquisition in an ombrotrophic peatland. Appl. Environ. Microbiol..

[71] Feng H., Ma M., Wang Z., Ma Y., Wang S. (2023). Diversity and community composition of carbon-fixing microbes along precipitation gradient in the Tibetan Plateau. Catena.

[72] Yabe S., Aiba Y., Sakai Y., Hazaka M., Yokota A. (2011). *Thermogemmatispora onikobensis* gen. nov., sp. nov. and *Thermogemmatispora foliorum* sp. nov., isolated from fallen leaves on geothermal soils, and description of *Thermogemmatisporaceae* fam. nov. and *Thermogemmatisporales* ord. nov. within the class *Ktedonobacteria*. Int. J. Syst. Evol. Microbiol..

[73] Guo X., Yin H., Liang Y., Hu Q., Zhou X., Xiao Y., Ma L., Zhang X., Qiu G., Liu X. (2014). Comparative genome analysis reveals metabolic versatility and environmental adaptations of *Sulfobacillus thermosulfidooxidans* strain ST. PLoS ONE.

[74] Hug L.A., Castelle C.J., Wrighton K.C., Thomas B.C., Sharon I., Frischkorn K.R., Williams K.H., Tringe S.G., Banfield J.F. (2013). Community genomic analyses constrain the distribution of metabolic traits across the Chloroflexi phylum and indicate roles in sediment carbon cycling. Microbiome.

[75] Wu C., Yan B., Wei F., Wang H., Gao L., Ma H., Liu Q., Liu Y., Liu G., Wang G. (2023). Long-term application of nitrogen and phosphorus fertilizers changes the process of community construction by affecting keystone species of crop rhizosphere microorganisms. Sci. Total Environ..

[76] Wang Q., Zhang Y., Zhang P., Li N., Wang R., Zhang X., Yin H. (2023). Nitrogen deposition induces a greater soil C sequestration in the rhizosphere than bulk soil in an alpine forest. Sci. Total Environ..

[77] Harcombe W.R., Riehl W.J., Dukovski I., Granger B.R., Betts A., Lang A.H., Bonilla G., Kar A., Leiby N., Mehta P. (2014). Metabolic resource allocation in individual microbes determines ecosystem interactions and spatial dynamics. Cell Rep..

[78] Schimel J., Balser T.C., Wallenstein M. (2007). Microbial stress-response physiology and its implications for ecosystem function. Ecology.

[79] Tan Q., Hao T., Gao S., Liu X., Wang G., Yu Q. (2020). Soil organic carbon turnover recovers faster than plant diversity in the grassland when high nitrogen addition is ceased: Derived from soil 14C evidences. Glob. Ecol. Conserv..

[80] Qiao L., Zhou H., Wang Z., Chen W., Li Y., Wu Y., Liu G., Xue S. (2024). Nine-year fertilization promoted C sink and mediated microbial nutrient utilization in alpine meadow soil aggregates. Appl. Soil Ecol..

[81] Liang C., Schimel J.P., Jastrow J.D. (2017). The importance of anabolism in microbial control over soil carbon storage. Nat. Microbiol..

[82] Smercina D.N., Bowsher A.W., Evans S.E., Friesen M.L., Eder E.K., Hoyt D.W., Tiemann L.K. (2021). Switchgrass rhizosphere metabolite chemistry driven by nitrogen availability. Phytobiomes J..

[83] Wang F., Gao Y., Li X., Luan M., Wang X., Zhao Y., Zhou X., Du G., Wang P., Ye C. (2024). Changes in microbial composition explain the contrasting responses of glucose and lignin decomposition to soil acidification in an alpine grassland. Sci. Total Environ..

[84] Weiße A.Y., Oyarzún D.A., Danos V., Swain P.S. (2015). Mechanistic links between cellular trade-offs, gene expression, and growth. Proc. Natl. Acad. Sci. USA.

[85] Feng X., Qin S., Zhang D., Chen P., Hu J., Wang G., Liu Y., Wei B., Li Q., Yang Y. (2022). Nitrogen input enhances microbial carbon use efficiency by altering plant–microbe–mineral interactions. Glob. Change Biol..

[86] Angst G., Mueller K.E., Nierop K.G., Simpson M.J. (2021). Plant-or microbial-derived? A review on the molecular composition of stabilized soil organic matter. Soil Biol. Biochem..

[87] Lavallee J.M., Soong J.L., Cotrufo M.F. (2020). Conceptualizing soil organic matter into particulate and mineral-associated forms to address global change in the 21st century. Glob. Change Biol..

[88] Liu J., Dai L., Chen Q., Guo X. (2024). Nitrogen addition favors terrestrial ecosystem carbon sink: A global meta-analysis. Sci. Total Environ..

