# Supplementary material for: Microbial Transcriptional and Metabolic Shifts Mediate Rhizosphere Organic Carbon Accumulation in Alpine Grasslands Following Six Years of Continuous Nitrogen Addition
Source: Microorganisms. 2026 Aug 17;14(8):1808. doi: 10.3390/microorganisms14081808 (PMC13515554; doi:10.3390/microorganisms14081808)
Supplement: Supplementary file 1 [file microorganisms-14-01808-s001.zip › microorganisms-4401590-supplementary.pdf]

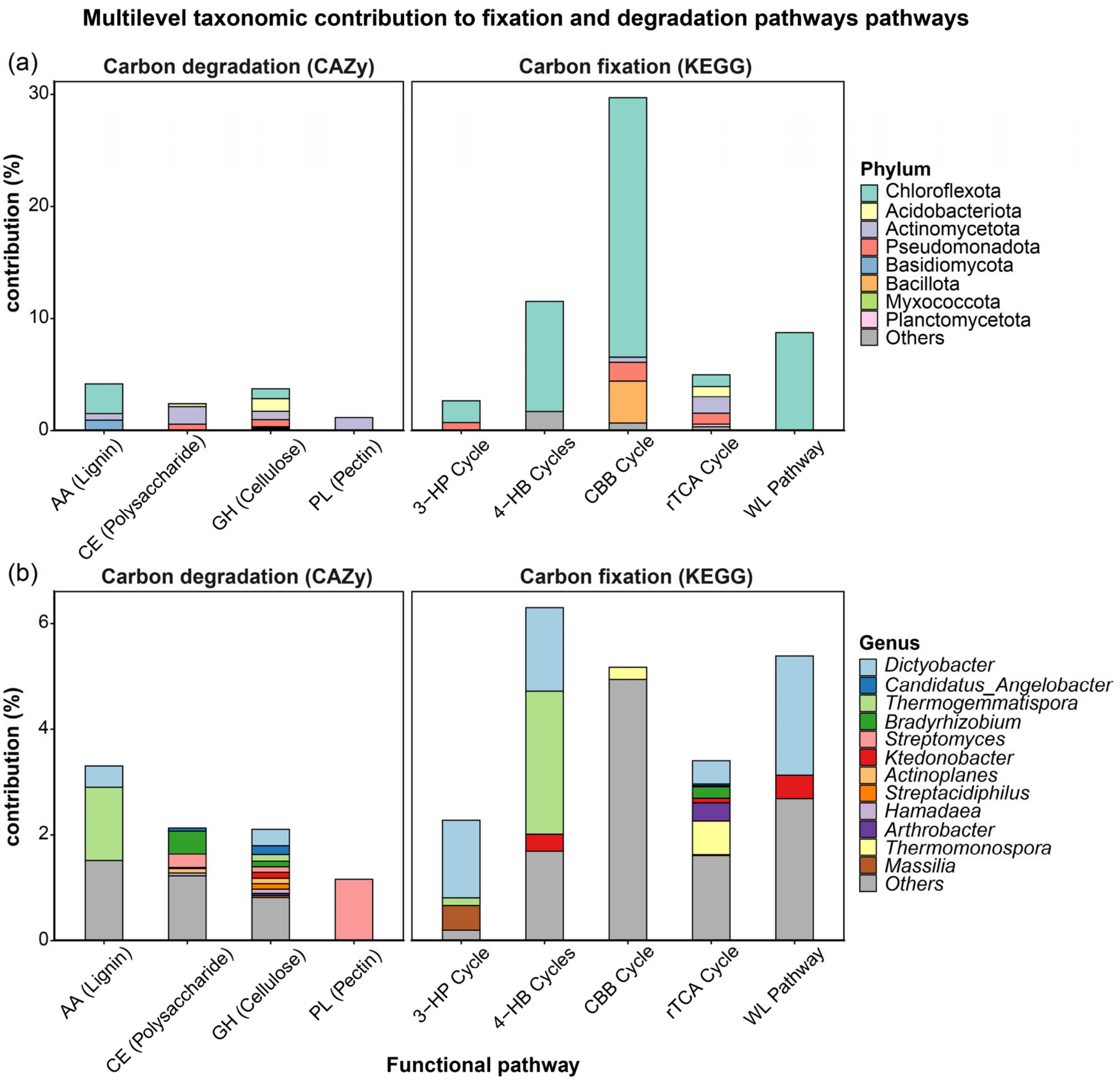

Figure S1. Multilevel taxonomic contributions to carbon fixation and degradation pathways. (a) The known contribution (%) of active microbial phyla to specific carbon degradation (annotated via the CAZy database) and carbon fixation (annotated via the KEGG database) pathways. **(b)** The known contribution (%) of active microbial genera to the corresponding functional pathways. In the stacked bar charts, different colors correspond to dominant specific taxonomic groups driving each metabolic process, while taxa with lower relative contributions are aggregated as 'Others'. **Pathway abbreviations:** CBB, Calvin-Benson-Bassham; *rTCA*, reductive tricarboxylic acid; WL, Wood-Ljungdahl; 3-HP, 3-hydroxypropionate; 4-HB, 4-hydroxybutyrate; GH, Glycoside Hydrolase; CE, Carbohydrate Esterase; AA, Auxiliary Activities; PL, Polysaccharide Lyase.

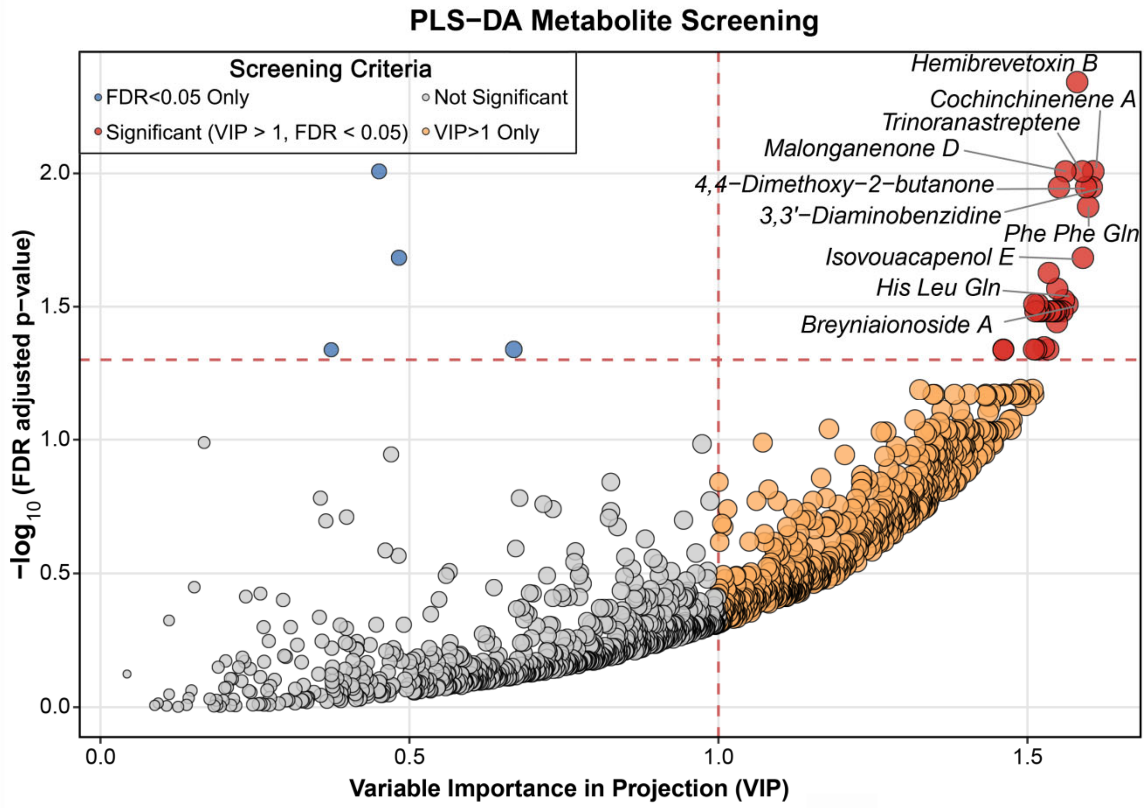

Figure S2. Identification of differential metabolites via partial least squares-discriminant analysis (PLS-DA). Red dashed lines denote the critical thresholds of Variable Importance in Projection (VIP) = 1.0 and false discovery rate (FDR) = 0.05. Red scatter points indicate significantly differential metabolites (VIP > 1.0 and FDR < 0.05). To optimize visual clarity, only the top 10 significantly differential metabolites, ranked by their VIP scores, are explicitly labeled.

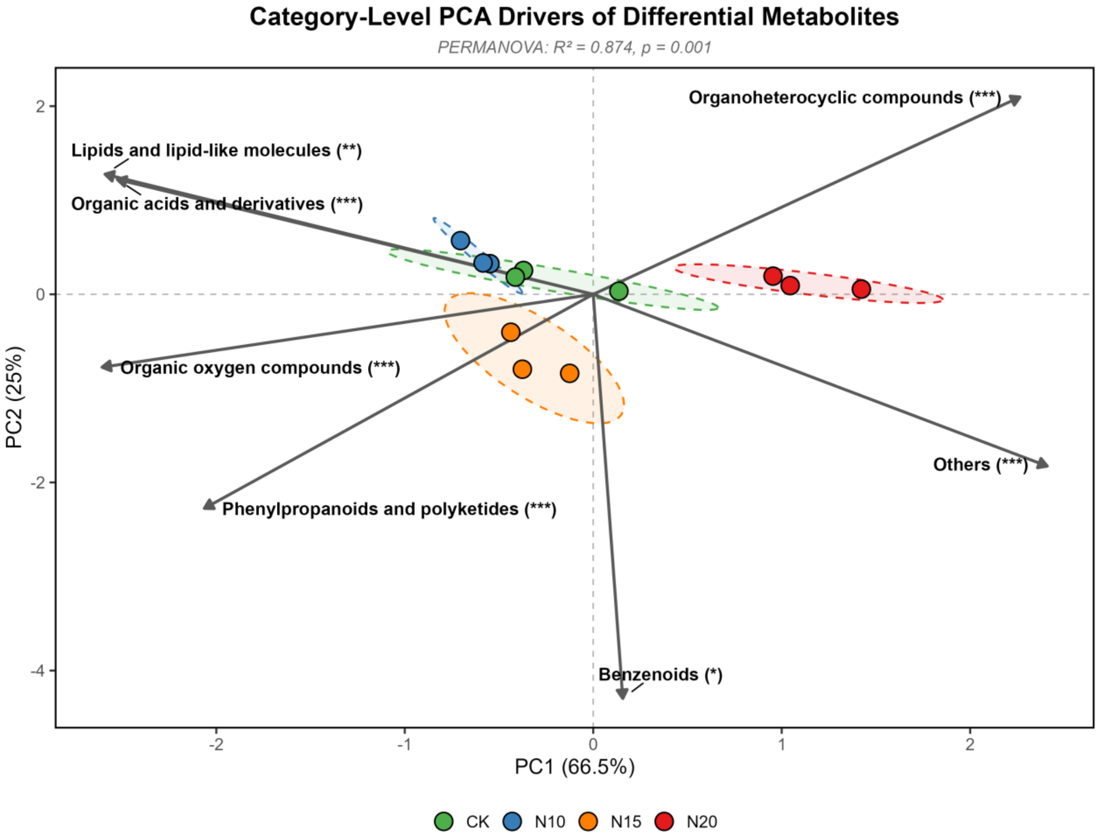

Figure S3. Principal component analysis (PCA) of rhizosphere differential metabolites categorized by metabolic superclasses. Colored scatter points represent individual samples across the continuous nitrogen (N) addition gradient (CK: 0, N10: 10, N15: 15, and N20: 20 kg N ha^{-1} yr^{-1}). Dashed ellipses denote the 95% confidence intervals for each treatment cluster. Asterisks indicate statistically significant differences in the abundance of specific metabolic superclasses across N treatments, as determined by one-way analysis of variance (ANOVA) (* $P < 0.05$, ** $P < 0.01$, *** $P < 0.001$).
